# 

**Published:** 1888-12

**Authors:** 


### NECROLOGY.

The deaths of Dr. George W. Keely, Treasurer of the American Association, and Dr. J. H. Prewitt, First Vice-President of the Southern Association, were reported, and memorial resolutions adopted.

Also for Drs. Stoddard Driggs, I. H. De Vore, Wm. Dutch, and C. P. Fitch, members of the American Association; and Drs. J. S. Franklin, H. M. Grant, and J. H. Cook, of the Southern Association.

---

### OBITUARY.

TO THE

### INDEPENDENT PRACTITIONER.

### VOL. IX.

### ORIGINAL COMMUNICATIONS.

Address of President R. R. Andrews.....527
Address of President W. F. Fundenberg.....411
Address of President H. C. Merriam.....
An Address on Congenital Deformities of the Mouth and Face. Roswell Park.....289
Anæsthesia—Physical and Psychical. B. A. R. Ottolengui.....183
An Attempt to Construct an Antiseptic Mouthwash. W. D. Miller.....175
Benefits—Dental and Otherwise. C. C. Barker.....572
Chromogenic Bacteria of the Human Mouth. Their Relation to the Different Colors of Decayed Dentine. W. D. Miller.....393
Clinic of Prof. Garretson, Hospital of Oral Surgery, Philadelphia. Robert S. Ivy.....17
Contributions to the History of Development of the Teeth. Carl Heitzmann and C. F. W. Bodecker.....1, 57, 113, 169, 225, 285, 344
Crown and Bridge-Work. F. T. Van Woert.....121
Dead and Diseased Teeth and their Treatment. E. S. Niles.....230
Dental Therapeusis, The Trend of. W. Xavier Sudduth.....398
Dental Education in Germany. W. D. Miller.....6, 66
Dental Resources. S. B. Palmer.....239
Eruption of the Permanent Teeth. C. N. Pierce.....456
Extraction of Molars, The. G. W. Weld.....75
Germ Theory of Dental Caries, The. George S. Allan.....505
Gas Furnaces and Enamel Fillings. W. H. Rollins.....301
Homœopathic Therapeutics in Dental Pathology. W. Irving Thayer.....636
Implantation of Human Teeth. Edward C. Kirk.....449
Implantation of Teeth, The. C. L. Curtis.....
Irregularities and Their Corrections. W. E. Magill.....465
My Way of Treating Nerve Cases. T. F. Chupein.....244

Oral Surgery. R. C. Brewster....125
Our Dental Literature. L. Ashley Faught....461
Past and Present Teachings in the use of Gold Foil. James Truman....404
Pathogenic Bacteria of the Human Mouth. W. D. Miller....281, 337
Personal Recollections of a Dentist of the Early Days. L. W. Bristol.... 12
Professional Patents. B. Holly Smith....621
Ptomaines. Charles Mayr....524
Relation of the Teeth and Palate to Vocalism, The. Thomas Fillebrown....557
Resection of the Superior Maxilla for Sarcoma. Herman Mynter....347
Root Canals Considered in Relation to Filling and Preparation of Artificial Substitutes. Louis Ottofy....566
Root Canals, Immediate Filling of. W. H. Potter....631
Social Culture for Dentists. W. F. Arnold....129
Treatment of Pulpless Teeth, The. George F. Cheney....520
Use of Copper Amalgam, The. Sidney S. Stowell....518

### REPORTS OF SOCIETY MEETINGS.

American Dental Association.... 19
American Dental Society of Europe....25, 87, 138
American and Southern Dental Associations....592
Central Dental Association of Northern New Jersey.... 91, 142, 257
Connecticut Valley and Massachusetts Dental Societies.... 539, 579, 660
Illinois State Dental Society....354, 415, 469
Ninth International Medical Congress, Washington, D. C., September, 1887, 21, 81, 132, 201, 253
New Jersey State Society....641
New York Odontological Society....97, 149
Pennsylvania State Dental Society....423, 484, 529
Pennsylvania State Dental Society Clinics....478
Southern Dental Association.... 29
The National Association of Dental Examiners....602

### EDITORIAL.

W. C. BARRETT, M.D., D.D.S. M.D.S.

Anæsthesia and Anæsthetics....152
Anæsthesia and Anæsthetics. No. II....264
A Dental vs. a Medical Congress....271
An Uncalled for Criticism.... 44
A Warning Instance....158
Bibliographical....46, 104, 158, 380, 500
Carbolic Acid as an Obtundent....157
Change of Address....211
Credit to Whom Credit is Due.... 45
Crowded Out....211
Dental Engine, The....267

Devitalizing Pulps ....269
Dogmatism ....268
Editorial Notices ....154
Fastidious Operating .... 46
Horsford's Acid Phosphate ....103
Immediate Root Filling .... 29
Matrices .... 98
"Mr. Bullin's Tribute to America." ....102
Mouthwashes ....209
Rather Crude ....101
Retrospective and Prospective ....668
The A. D. S. E. Report .... 43
The Independent Practitioner ....102
The Triumph of Law ....210
To Whom It May Concern ....211
Valedictory ....371
Welding of Gold ....156

W. X. SUDDUTH, M.D., D.D.S., F.R.M.S.

A Disappointment ....500
American and Southern Dental Societies, Clinics and Exhibits ....670
An Enjoyable Affair, ....613
Announcements ....379, 497, 616
An Explanation ....615
Bibliographical ....443, 676, 677, 678
Business Meetings, American and Southern Dental ....673
Complimentary ....615
Conservatism and Radicalism in Matters of Dental Education ....437
Connecticut Valley and Massachusetts Union Meeting ....548
    Downer's Landing and the Clam Bake ....550
    The Exhibit ....551
    The Clinics ....553
Dental Department, University Iowa ....614
Dentistry a Specialty in Medicine ....433
Dental Education.—Resolutions ....441
"Editorial Boasting." ....498
Elections, American and Southern Dental Societies ....678
National Association of Dental Faculties ....376
National Association of Dental Faculties ....606
    A Body Without Authority .... 607
    A Graded Course ....609
    An Earnest Meeting ....611
    The Way Out ....613
Necessity for a Higher Standard of Education ....435
Necrology ....674

New Syndicate, The............373
Obituary............674
Probable Manner of Attachment of Implanted Teeth............493
The Prognosis............495
A Word of Caution............496
Saccharine............546
Physical Appearance............546
Tests for............547
Special Announcement............497
Twentieth Annual Meeting of the Pennsylvania State Society............439
Valedictory............667
Western Dental Journal, Criticism of............378

CURRENT NEWS AND OPINION.

............51, 106, 162, 212, 271, 328, 384, 442, 501, 554, 616, 679

---

### PROSPECTUS FOR 1889.

THE

### International Dental Journal,

SUCCESSOR TO THE

### INDEPENDENT PRACTITIONER.

PUBLISHED BY THE

International Dental Journal Company:

**LOUIS JACK,** *President*, Philadelphia.

**BENJAMIN LORD,** *Vice-President*, New York.

**C. U. PEIRCE,** *Treasurer*, Philadelphia.

**GEO. S. ALLAN,** *Secretary*, 51 W. 37th St., New York.

**W. X. SUDDUTH, M.D., D.D.S., F.R.M.S.,**

Editor and Business Manager, 1215 Filbert St., Philada.

### The Independent Practitioner—Vol. IX.

### PUBLISHED BY DENTISTS FOR DENTISTS.

### PROSPECTUS.

The Independent Practitioner has assumed a more important role as the organ of the International Dental Journal Company. The promises of the New York Dental Journal Association of making Volume IX better than any former volumes have been well carried out. The scope of the Journal has been enlarged, and its circle of influence greatly increased. We still continue the magnificent offer of Dr. Stowell's work on the *Microscopic Structure of a Human Tooth*, which is published at the moderate price of six dollars each. We have made such arrangements with the publishers that we can furnish it in connection with a subscription to **The Independent Practitioner for One Dollar and Fifty Cents. That is, to every one who will send in Four Dollars we will send the Independent Practitioner one year and a copy of this beautiful and useful atlas.**

This offer is intended only for subscribers in America. Foreign residents within the postal union must enclose fifty cents extra for prepayment of postage upon the Journal. Our regular rate of subscription to such is three dollars per annum.

As the book absolutely costs more than the sum for which it will be furnished, the money must accompany the subscription. Nor will the book be sold separately. It will only be sent in connection with a subscription to this journal.

The book will be sent either by mail or express. If it is desired that it should be sent by mail, the postage (twenty-five cents) should be sent. All remittances should be sent to the Editor and Business Manager, Dr. W. X. Sudduth, 1215 Filbert Street, Philadelphia, Pa. Postal orders, Postal Notes, or New York Drafts are the safest and most convenient.

The Independent Practitioner has been transferred to a new and enlarged syndicate—no material change is, however, to be made in the form of the journal until the close of the present volume. We hope to hold all our old subscribers, and obtain many new ones. You may hand your subscription to any one of the stockholders, a list of whose names is here appended.

GEO. S. ALLAN,
R. R. ANDREWS,
H. A. BAKER,
W. C. BARRETT,
A. P. BEALE,
S. T. BEALE,
C. S. BECK,
G. V. BLACK,
E. A. BOGUE,
C. F. W. BODECKER,
W. G. A. BONWILL,
C. A. BROCKETT,
EDWARD C. BRIGGS,
THOS. BRADLEY,
ALBERT H. BROCKWAY,
A. E. BROWN,
WM. CARR,
G. F. CHENEY,
DWIGHT M. CLAPP,
JOHN D. CODMAN,
CHAS. D. COOK,
J. N. CROUSE,
EDW. T. DARBY,
D. M. DAVIDSON,
WM. H. DWINELLE,
CHAS. J. ESSIG,
J. N. FARRAR,
T. FILLEBROWN,
W. B. FINNEY,
T. A. FLETCHER,
M. W. FOSTER,
CHAS. E. FRANCIS,
W. F. FUNDENBERG,
W. H. FUNDENBERG,
E. C. FUNDENBERG,
A. H. GILSON,
E. S. GAYLORD,
J. P. GERAN,
S. H. GUILFORD,
JOHN J. HART,
O. E. HILL,
J. F. P. HODSON,
J. MORGAN HOWE,
ROBERT HUEY,
LOUIS JACK,
C. A. KINGSBURY,
EDW. C. KIRK,
C. R. E. KOCH,
W. T. LA ROCHE,
W. K. LEECH,
D. F. A. LEVY,
WILBUR F. LITCH,
J. BOND LITTIG,
BENJAMIN LORD,
GEO. W. LOVEJOY, (Canada),
JOHN S. MARSHALL,
JAMES McMANUS,
CHARLES McMANUS,
E. V. McLEOD,
H. J. McKELLOPS,
DAN'L. NEAL McQUILLAN,
HORATIO C. MERIAM,
W. D. MILLER,( Berlin),
W. E. PAGE,
S. B. PALMER,
C. B. PARKER,
D. D. PEABODY,
S. G. PERRY,
C. N. PEIRCE,
CHAS. E. PIKE,
W. A. PHREANER,
W. E. PINKHAM,
W. H. POTTER,
H. C. REGISTER,
Z. T. SAILER,
L. D. SHEPARD,
EUGENE H. SMITH,
B. HOLLY SMITH,
S. G. STEVENS,
W. X. SUDDUTH,
AMBLER TEES,
J. D. THOMAS,
JAMES TRUMAN,
WM. H. TRUEMAN,
F. F. VAN WOERT,
W. W. WALKER,
I. T. WARDWELL,
C. S. WARDWELL,
G. W. WARREN,
T. WATERS,
GEO. W. WELD,
JULIUS G. W. WERNER,
JACOB L. WILLIAMS,
R. B. WINDER,
C. A. WOODWARD,
J. A. WOODWARD.

